# Does long-term care insurance reduce the disability among middle-aged and older adults? Evidence from China

**DOI:** 10.1186/s12889-023-16057-0

**Published:** 2023-06-13

**Authors:** Na Cao, Tong Shi, Chaoping Pan

**Affiliations:** 1 School of Public Health and Management, Wenzhou Medical University, Wenzhou Medical University Chashan Campus, Wenzhou City, 325035 Zhejiang Province China; 2 School of Public Health, Wuhan University, Wuhan City, Hubei Province China

**Keywords:** Older adults, Disability, Long-term care insurance, Difference-in-differences method, China

## Abstract

This study aimed to examine whether the implementation of Long-Term Care Insurance (LTCI) policy could reduce the disability among middle-aged and older adults in China, and to test the heterogeneity of the effects. Data came from four waves of the China Health and Retirement Longitudinal Study (CHARLS, 2011 to 2018). The Difference-In-Differences (DID) method and the panel data fixed effect model were used to estimate the effect of implementation of LTCI policy on disability among individuals aged 45 years and above. The LTCI policy had a positive impact on reducing disability among middle-aged and older people. Females, younger adults, city dwellers, and individuals living alone benefited the most from LTCI policy. The results provided empirical evidence for the implementation of LTCI policy in China and other similar countries as China. The implementation of LTCI policy should also pay more attention to inequity of the effects on reducing disability among different demographic groups.

**Supplementary Information:**

The online version contains supplementary material available at 10.1186/s12889-023-16057-0.

## Introduction

### Background

Disability refers to the loss or limitation of the capacity to carry out daily tasks [1, 2]. Over the last half-century, advances in medical care have extended life expectancy, resulting in a greater number of people living with disabilities during their later years [3]. Above 50% of older adults among OECD countries reported having at least one limitation in their daily activities, and 17% reported severe limitations in 2017 [4]. China is also facing an ageing population and the high prevalence of chronic disease [5, 6], which may implicate more disabled older adults in the future. China already had more than 40.6 million disabled or partially disabled older adults, accounting for 18.3% of the total ageing population, and the number may increase to 65 million in 2030 [7]. Rising number of aged people, empty-nest families, and the overburdened health care system make it difficult for society to take care of older adults living with a disability. So it is necessary to reduce disability to lower social burden and improve the wellbeing among older adults.

To tackle these challenges, the city of Qingdao in Shandong province became the pioneering city in China to implement Long-term Care Insurance (LTCI) in 2012. The initial guideline for the LTCI policy in China intended to be implemented in 15 pilot cities in 2016 (see Table 1a of suppmentary appendix). To further promote the implementation of LTCI policy, the government added 14 new pilot cities to the initial 15 pilot cities in September 2020 (see Table 1b of suppmentary appendix).

Overall, LTCI in pilot cities paid eligible individuals about 70% of the cost for Long-Term Care (LTC) in the recorded categories in LTCI [8]. LTCI covered those enrolled in the Urban Employees Basic Medical Insurance (UEBMI), Urban Resident Basic Medical Insurance (URBMI) or Urban-Rural Residents Basic Medical Insurance (URRBMI) in seven pilot cities, whereas in eight pilot cities, LTCI covered individuals enrolled in UEBMI (see Table 1a of suppmentary appendix). The service forms generally included institutional care and home care for the disabled, which mainly provided medical, rehabilitation and nursing care services.

A significant number of OECD countries have yet to establish Long-term Care Insurance (LTCI) programs despite the context of population aging and increasing rates of disability [9]. To further enhance LTCI policy, it is crucial to understand how its execution will affect disability of the insured. Our study aimed to examine the effects of implementation of LTCI policy on reducing disability among middle-aged and older adults in China, and to test the heterogeneity of the effects among different groups, which may provide evidence for the implementation of LTCI policy in China and other aging countries.

### Theoretical framework

The association between LTCI and disability can be explained on the basis of disablement process model. It proposes that both intra- and extra-individual elements may have an impact on the process of disablement [10]. Intra-individual factors include the demographic factors such as age, sex and so on. While extra-individual factors indicate the environmental factors such as social supports, nursing home policies, insurance coverage and so on [10, 11]. As an extra-individual factor, prior studies were indicative of a strong preference for LTCI in an ageing society because LTCI could significantly affect the health of eligible insured persons [12–15]. The mechanisms behind this may lie in the fact that LTCI could meet healthcare service needs and reduce healthcare burden of the disabled for current care recipients [16–18]. For non-recipients covered by LTCI, LTCI may improve their health by reducing psychological burdens from expected long-term care needs or relieving actual care burdens for family caregivers of current care recipients [16].

### Literature review and hypothesis

Previous studies suggested that LTCI had positive health benefits on middle-aged and older adults [16, 18–20], and some studies have also been conducted in the context of China. For example, Ishibashi et al. indicated that using home help services had a positive effect on maintaining IADL function [20]. Lei et al. found that LTCI coverage could improve self-reported health and reduce one-year mortality risk among older adults in China [16]. Fan et al. found a beneficial effect of LTCI coverage on self-rated life satisfaction among disabled Chinese older adults [18]. Nevertheless, certain studies have also suggested that the implementation of LTCI policy may not yield health-related benefits [21, 22]. For example, Nanako et al. discovered that LTCI had no impact on IADL disability and self-rated health [21]. Kim et al. found that the LTCI did not affect the mortality among older adults [22]. Overall, the impacts of LTCI on health were inconclusive, and there was still a lack of systematic research on how LTCI affected disability. Based on previous studies, we proposed the following hypotheses:

#### Hypothesis 1a

The implementation of LTCI policy reduced the disability among middle-aged and older adults in China.

#### Hypothesis 1b

The implementation of LTCI policy did not influence the disability among middle-aged and older adults in China.

As LTCI paid the insured person according to their disability levels without considering other factors of the insured (e.g., social resources), it is still not known which groups may gain more health benefits from LTCI policy. Previous evidences suggested that LTCI might serve as a replacement for informal social supports because it allowed those with less social capital to access more health care services [22–24]. In other words, when the disabled lacked social supports, LTCI could make up the disadvantage of the informal social supports and social resources (e.g., family supports). Meanwhile, other research has indicated that if Long-term Care Insurance (LTCI) offers inadequate subsidies to the underprivileged, they may still face difficulties in accessing healthcare services [15, 23, 25]. Most scholars focused on age, gender, education, residence, living arrangement inequity in health profits from the LTCI policy [15, 18, 22, 25–27], but few studies focused on the heterogeneity of the inequitable benefits of reducing disability from LTCI policy. According to literature review, we proposed the second hypothesis:

#### Hypothesis 2

The impact of LTCI on disability among older adults showed variations depending on age, gender, education, residence and living arrangement.

## Methods

### Study population and measurements

The dataset was derived from the 2011–2018 waves of the China Health and Retirement Longitudinal Study (CHARLS). CHARLS is a longitudinal survey that designed to represent the residents in mainland China aged 45 and older, with no upper age limit. The national baseline survey was conducted in 2011/2012, with subsequent waves in 2013, 2015, and 2018. To ensure sample representativeness, the CHARLS baseline survey covered 150 counties/districts and 450 villages/urban communities across the country, involving individuals in 10,257 households at the baseline, reflecting the middle-aged and older Chinese population. Further details about CHARLS can be found in the study by Ferraro et al. [28]. This study specifically targeted respondents who were 45 years of age and older. The sample characteristics are shown in Table 2a and Table 2b of supplementary appendix. Regarding attrition, we opted to exclude the data with missing values directly from our analysis. However, we also utilized the Multiple Imputation (MI) method to replace missing data and re-estimated the results, which are presented in the sensitive analysis part of the paper.

### Dependent variables

Disability status was evaluated through measures of Activity of Daily Living (ADL), Instrumental Activity of Daily Life (IADL), and Functional Limitations (FL). ADL was formed by six items (bathing, dressing, toileting, indoor transferring, continence, and feeding). IADL was measured by eight items (managing money, using the phone, taking medications, preparing hot meals and shopping for groceries). FL was measured at each wave by four items: (a) stand up from sitting in a chair, (b) hand behind neck, (c) hand behind the lower back, (d) able to pick up a book from the floor. Each item on ADL and IADL was accessed on two grades: no difficulty (0) and some difficulty/inability to perform the task (1). Each item on FL was accessed on three grades: no difficulty (0), some difficulty (1) and inability to perform the task (2). Thus, the total scores of ADL, IADL and FL ranged from 0 to 6, from 0 to 5 and from 0 to 8.

### Independent variables

Participants who attended UEBMI, URBMI or URRBMI in pilot cities were covered by LTCI. The details of participants of LTCI in each pilot city are provided in Table 1a of suppmentary appendix. Participants taking part in LTCI were coded as “1” and otherwise were coded as 0.

### Covariates

The selection of covariates was based on the disablement process model. According to previous studies [10, 11, 29, 30], we included gender, age, education, residence and health behaviors as intra-individual factors, and living status, basic medical insurance were included as extra-individual factors. Disablement process factors included chronic disease and depression. Gender was coded as female (1) and male (0). Age was measured at baseline, and was divided into younger adults (below 60 years) and older adults (60 years and above). Education ranged from illiterate (1) to college and above (8). Living status was coded as not living alone (1) and living alone (0). Health behaviors included as smoking (frequency of smoking last year) and drinking (frequency of drinking last year). Residence was included as rural (0) and urban (1). Basic medical insurance was included as having basic medical insurance (1) or not having (0). The measure of chronic disease was derived from self-reported health conditions, with a score ranging from 0 to 12. This score encompassed various conditions such as hypertension, diabetes, and others. Depression was measured by the CESD-10 scale and composed of ten questions including “feeling depressed”, “feeling lonely”, etc. [31]. The total score of depression ranged from 0 to 30, with higher scores indicating the more severe perceived depression. For the purpose of heterogeneity analysis, the time-variant variables were categorized based on their baseline characteristics. Additionally, when these variables were considered as covariates, they were included in the analysis as time-variant variables.

### Statistical analysis

To evaluate the impact of LTCI implementation on disability, the study employed the Difference-in-Differences (DID) method, which was analyzed using a linear panel data fixed-effect model. To account for the skewness of the outcome variables and improve the robustness of model assumptions, the study utilized a Huber-White sandwich variance estimator in all analyses. The base regression model was set as follows:

1 $${y}_{it}=\alpha +\beta {Treat}_{ij}\times {Post}_{it}+\delta {X}_{it}+{\tau }_{t}+{\omega }_{i}+{\epsilon }_{it}$$

In the model, ${y}_{it}$ represents ADL, IADL, or FL. The variable ${Treat}_{ij}$ indicates weather individuals were covered by LTIC. If the population was covered by LTCI, then${Treat}_{ij}$ was coded as 1, otherwise, it was coded as 0. The variable ${Post}_{it}$ indicates whether the LTCI policy was implemented locally. It was set to 0 before implementation and 1 after implementation (The implementation time of local LTCI was based on Table 1a of suppmentary appendix). The coefficient $\beta$ represents the net effect of the implementation of LTCI on disability, which is the main focus of the study. The variables ${X}_{it}$ are a series of time-variant variables, including living status, residence, basic medical insurance, smoking, drinking, chronic disease, and depression. ${\tau }_{t}$ represents the time fixed effect, ${\omega }_{i}$ represents individual fixed effects, and ${\epsilon }_{it}$ represents the error term.

## Results

The demographic characteristics of the treated and control groups are shown in Table 2a and Table 2b of the supplementary appendix, while the number of older adults who had ADL, IADL and FL scores of more than 1 in treated the group is presented in Table 2c of supplementary appendix.

### Effects of implementation of LTCI on disability

Table 1 shows the beneficial effects of LTCI implementation on reducing disability. The impacts of LTCI on IADL, ADL, and FL disabilities were 0.072, 0.279, and 0.830, respectively, which indicated that the implementation of LTCI policy could reduce the future development of IADL, ADL, and FL scores among older adults on average by 0.072, 0.279 and 0.830 points, though the impact of LTCI on IADL score was insignificant.

**Table 1:** Effects of implementation of LTCI on disability

Effects	IADL	ADL	FL
DID	-0.072(0.106)	-0.279**(0.101)	-0.830***(0.186)
Living status	-0.044(0.045)	0.010(0.042)	-0.036(0.059)
residence	0.012(0.033)	0.043(0.034)	0.038(0.057)
Basic medical insurance	-0.020(0.025)	-0.020(0.025)	-0.005(0.037)
Smoking	-0.001(0.001)	-0.001(0.001)	-0.001(0.001)
Drinking	-0.001(0.004)	-0.007(0.004)	-0.004(0.007)
Chronic disease	0.034***(0.012)	0.045***(0.011)	0.060***(0.016)
Depression	0.027***(0.001)	0.024***(0.001)	0.048***(0.002)
Individual fixed effect	control	control	control
Time fixed effect	control	control	control

Note: *** p < 0.01, ** p < 0.05, * p < 0.1; Robust standard errors are in parentheses.

### Heterogeneity analysis

Table 2 displays the effects of LTCI on disability by different demographic characteristics. In terms of gender, LTCI could significantly reduce FL and ADL scores by 0.571 and 0.151 points for males, and 1.170 and 0.443 points for females.

**Table 2:** Effects of implementation of LTCI on disability by different demographic characteristics

Dependent variables	Group variables	DID	Dependent variables		Group variables	DID
IADL	Male	-0.064(0.037)	IADL		Lower education	-0.049(-0.074)
	Female	-0.071(0.247)			Higher education	-0.039(-0.06)
ADL	Male	-0.151***(0.061)	ADL		Lower education	-0.207**(-0.081)
	Female	-0.443**(0.216)			Higher education	-0.100**(-0.042)
FL	Male	-0.571***(0.133)	FL		Lower education	-0.637***(-0.162)
	Female	-1.170***(0.385)			Higher education	-0.052(-0.131)
IADL	Younger	-0.248*(0.056)	IADL		LA	-0.086(-0.117)
	Older	-0.076(0.303)			NLA	-0.028(-0.054)
ADL	Younger	-0.396*(0.227)	ADL		LA	-0.200(-0.054)
	Older	-0.317(0.205)			NLA	-0.151***(-0.049)
FL	Younger	-1.061***(0.388)	FL		LA	-0.952***(-0.286)
	Older	-1.003**(0.396)			NLA	-0.297**(-0.117)
IADL	Urban	-0.061(-0.065)				
	Rural	-0.015(-0.247)				
ADL	Urban	-0.178***(-0.057)				
	Rural	-0.100(-0.087)				
FL	Urban	-0.431***(-0.131)				
	Rural	-0.177(-0.175)				

Note: Covariates, individual fixed effect, time fixed effect were all controlled; *** p < 0.01, ** p < 0.05, * p < 0.1; Robust standard errors are in parentheses; NLA = not living alone; LA = living alone; When regressing urban and rural samples, covariates did not include residence; When regressing LWS and NLWS samples, covariates did not include living status; Individuals above average education level were treated as higher education and otherwise were treated as lower education

We classified individuals aged under 60 as younger adults, and those aged 60 and above as older adults. The results indicated that LTCI significantly reduced FL, ADL and IADL scores among younger adults by 1.061, 0.396 and 0.248 points, respectively. However, LTCI could only significantly affect FL score among older adults.

LTCI could reduce FL and ADL scores among urban people by 0.431 and 0.178 points, respectively. While LTCI could not impact the physical function among rural people.

In heterogeneity analysis, we divided the education categories into lower education (primary school and below) and higher education (above primary school). The results showed individuals with lower education could benefit from LTCI for ADL and FL scores by 0.207 and 0.637 points, respectively. While for individuals with higher education, LTCI could only reduce ADL score by 0.1 point.

Individuals living alone experienced a reduction in their FL score by 0.952 points, indicating a potential benefit of LTCI. In contrast, for individuals who did not live alone, LTCI demonstrated favorable effects in reducing both ADL and FL by 0.151 and 0.297 points, respectively. The details are shown in Table 2. For differences that could not be judged by subgroup significance, we used Wald chi-square tests to compare the effect sizes in subgroups. Our findings indicated that individuals living alone derived greater benefits from LTCI in terms of reducing FL, compared to those who did not live alone (χ^2^ = 4.49, p < 0.05). The aforementioned results suggested that LTCI had a more significant impact on younger adults, urban residents, and individuals with lower levels of education.

### Sensitive analysis

#### Robustness and placebo tests

**Fig. 1:**
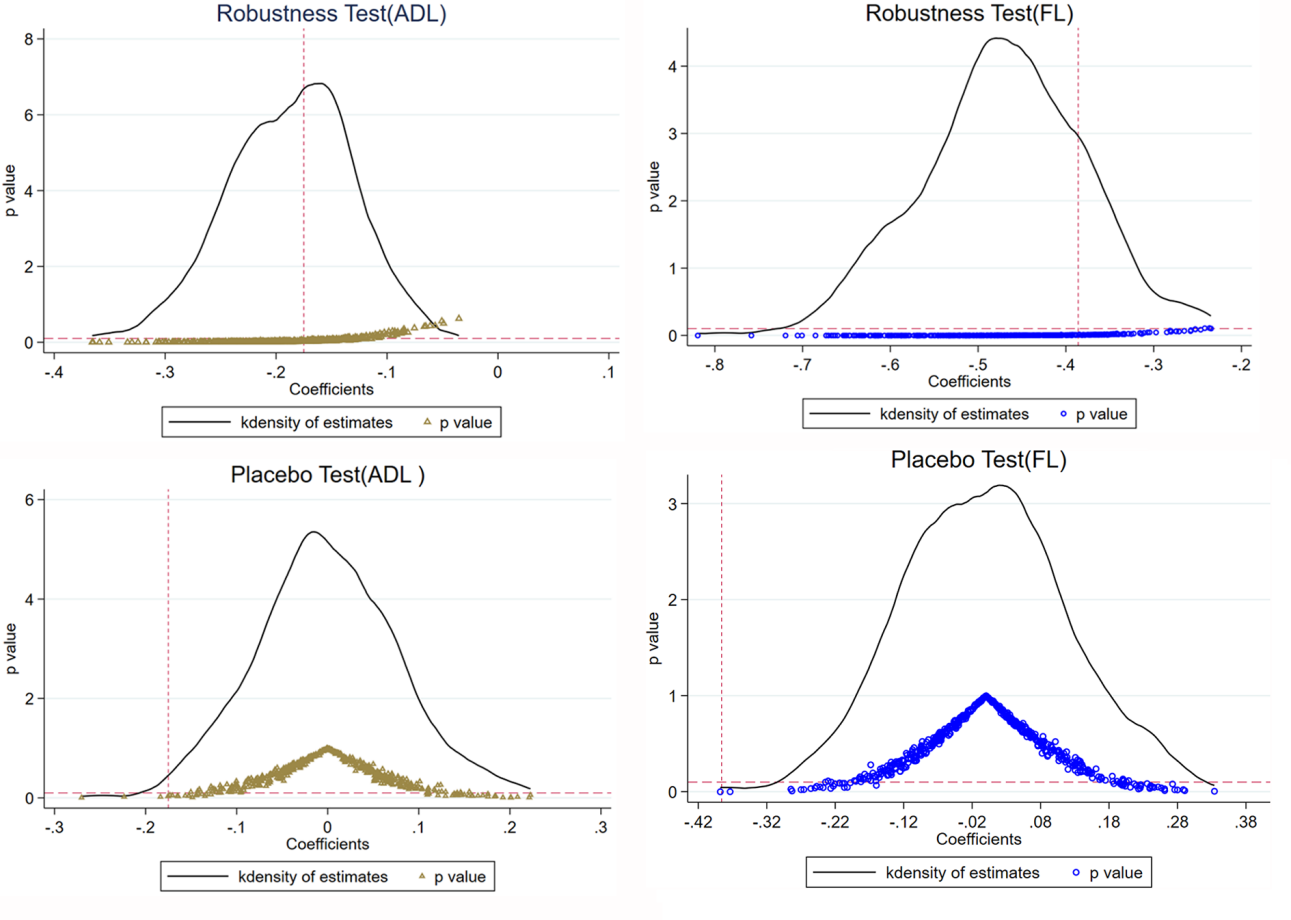
Results of the estimation adjusting proportion and placebo tests for 500 times

While we observed positive effects of implementing LTCI in reducing disability, it is important to acknowledge that these results may be influenced by other policies or random occurrences. The original treated group remained unchanged, while a new control group consisting of 300 randomly selected samples was created for the purpose of conducting a robustness test. This was done to address the notable disparity in proportions between the treated and control groups. After 500 iterations of the regression process using Eq. (1), the density distribution of the regression coefficient is shown in Fig. 1. It could be found that the coefficient of the benchmark regression (vertical dotted line) was close to the median of the density functions of the two graphs. Nearly all of the regression coefficients’ *p* values were below 0.1. This finding demonstrated that other policies and the relatively small sample size of the treated group did not have a significant influence on the conclusions drawn in the paper.

To further investigate the possibility of accidental findings, the study conducted a placebo test by randomly selecting 500 individuals as a treated group from the dataset. This analysis specifically focused on examining the effects on both ADL and FL. Black solid lines represented the kernel density estimation and the circles represented the corresponding *p* values. The estimates for both FL and ADL deviated significantly from the real estimates (-0.386 and − 0.175, respectively) and most *p* values were also greater than 0.1, indicating that the results were unlikely to be produced accidentally or to be influenced by other policies. Details are shown in Fig. 1.

### PSM-DID method, exogenous test and multiple imputation method

To ensure the parallel time trends between the control and treated groups, we employed the PSM-DID approach in this section to regulate the imbalanced distributions of the variables. The samples in 2011 and 2018 were retained. First, the PSM method was used to match the samples; second, the successfully matched samples were retained; finally, the matched samples were regressed according to Eq. (1). The specific results are shown in the upper part of Table 3, which supported the original conclusion shown in Table 1 and confirmed the robustness of our results.

**Table 3:** Effects of implementation of LTCI on disability by using PSM-DID method, exogenous test and multiple imputation methods

Effects	IADL	ADL	FL
Using PSM-DID method			
DID	-0.086(0.150)	-0.298**(0.080)	-0.428*(0.233)
Observations	16,915	11,365	11,365
Using 14 new pilot cities as control groups			
DID	-0.045(0.057)	-0.117**(0.058)	-0.364***(0.122)
Observations	2005	2045	2057
After filling in missing values			
DID	-0.055(0.049)	-0.169**(0.047)	-0.386***(0.107)
Observations	40,715	41,241	41,358

Note: *** p < 0.01, ** p < 0.05, * p < 0.1; Robust standard errors are in parentheses

K-nearest neighbor matching was performed on the selected treated and control groups, and K value was taken as 3. When the absolute normalized bias value of the matched variable was less than 20, the matching estimate was considered reliable. The results indicated that nearly all of the absolute normalized bias values of the matched variables were almost less than 20 after PSM (see in Table 3a-c of the supplementary appendix). At the same time, the t-statistic values after matching were not significant, indicating that the covariates were balanced after matching.

To mitigate the potential influence of chance findings, the study utilized the 14 newly designated pilot cities, which implemented the LTCI policy in 2020, as a control group. As the 14 new pilot cities had a similar economic development level, they may have comparable trends in disability to the 15 former pilot cities. The results showed that IADL, ADL, and FL scores decreased 0.045, 0.117 and 0.364 points after implementation of LTCI policy (shown in the middle part of Table 3), which was similar to the results shown in Table 1.

As for attrition, we deleted missing data directly, which may cause bias in our results. So we used the multiple imputation methods to replace the missing values due to intermittent missing. And we found similar results shown in Table 1, which were shown in the lower part of Table 3.

## Discussions

In response to the rapid aging population and the rising rates of disability among older adults, numerous countries including Japan, Singapore, the United States, and China have implemented LTCI policy. In order to enhance policy implementation, it was crucial to assess whether the implementation of LTCI policy could effectively reduce disability. However, there has been a scarcity of studies evaluating the impact of LTCI on disability outcomes. Some studies found that LTCI coverage could improve self-reported health and self-rated life satisfaction, and lower the risk of one-year mortality among Chinese older adults [16, 18]. Our results added the literature by demonstrating that LTCI policy could reduce disability in the context of Chinese society, and the results substantiated hypothesis 1a. The results could be explained by the factors that LTCI could meet health care service needs, relieve the strain of medical care on the disabled [16–18], reduce psychological burdens from the expected long-term care needs, and relieve actual care burdens for non-recipients covered by LTCI [16]. The beneficial effects of LTCI on disability can provide support for the implementation of LTCI policy in China and other aging countries.

Our study included some key baseline demographic factors of disablement process model as variables for heterogeneity analysis. The results found that the implementation of LTCI policy mattered more for younger adults, urban dwellers and individuals with lower education, which supported our second assumption.

Individuals with lower education benefited more from LTCI, which was in line with other studies [16, 27]. One possible explanation is that the LTCI policy, as a formal social support mechanism, may assist in compensating for the limited social resources available to individuals with lower levels of education [23, 32–34]. Our study also found that LTCI was more beneficial for younger adults. However, a previous study conducted in China found LTCI had a greater effect on inpatient care and health expenditures among older adults [4]. Our results may be explained by poor health resilience among older adults when taken together with that study’s findings [30]. Older adults may be less likely to recover from disability than younger adults, which could make LTCI less beneficial in reducing disability among older adults even though they may use more long-term care services than younger adults.

Despite the fact that rural areas had a higher rate of severe disability than urban areas in China [35], our study did not find evidence that rural residents could benefit from implementation of LTCI policy. Rural residents may have less access to long-term care services when they are disabled compared to urban residents due to unequal health care resource distribution between rural and urban areas, which made rural residents less likely to benefit from LTCI policy [35]. This indicates that there is a need for more long-term care services and resources in rural areas. Our findings suggested that the demographic factors including age, residence and education may influence the health consequences of LTCI policy. Therefore, the implementation of LTCI policy should pay greater attention to these demographic factors to ensure that all individuals have equal access to long-term care services regardless of their demographic characteristics.

### Limitations

Our study had some limitations. Firstly, the variables were based on self-reported surveys, which may have introduced bias. However, self-reported data (e.g., ADL, IADL and FL) are commonly used in disability research among older adults and may reflect personal status interacting with the real world more accurately [36]. Secondly, we were unable to determine the number of respondents in our sample who genuinely benefited from LTCI because disability assessments varied throughout pilot locations. We countered that the impacts might have been diminished by individuals who were not eligible for the benefits, and that the effects might be stronger than anticipated. Thirdly, despite incorporating control factors based on the disablement process model, it was not feasible to include all time-variant variables that could potentially influence disability. This limitation may have introduced bias into the results. Finally, LTCI may have different effects on disability between institutional care and home care. Unfortunately, due to the limitation of data, we were unable to analyze the differences in the role of LTCI in these two contexts. So, further studies are still required.

## Conclusion

This study contributes to the existing research in three respects: Firstly, a large four-wave national representative longitudinal dataset was used, which enabled better investigation of how the implementation of LTCI policy affected disability among middle-aged and older adults. Secondly, to the best of our knowledge, few studies focused on the effects of LTCI on disability. Our study provides a compelling argument for exploring the effects of LTCI on disability. The paper found the beneficial effect of LTCI policy on reducing disability, along with the observed heterogeneity across different demographic groups. These findings provide evidence for the feasibility of introducing LTCI in other similar aging countries. Thirdly, we employ a policy experiment by combined DID method with the linear panel data fixed-effect model to identify the effects of LTCI on reducing disability, potentially avoiding the endogenous problems and increasing the robustness of our study. To ensure that the results were not caused by selection bias or other policies, we performed a series of robustness tests, which showed the beneficial effects of LTCI on disability were less likely to be affected by selection bias or other policies.

## Electronic supplementary material

Below is the link to the electronic supplementary material.

Supplementary Material 1

## Data Availability

The dataset used in this paper are publicly available in http://charls.pku.edu.cn/en/.
