# Supplementary material for: Does long-term care insurance reduce the disability among middle-aged and older adults? Evidence from China
Source: BMC Public Health. 2023 Jun 13;23:1138. doi: 10.1186/s12889-023-16057-0 (PMC10265914; doi:10.1186/s12889-023-16057-0)
Supplement: Supplementary file 1 — Supplementary Material 1 [file 12889_2023_16057_MOESM1_ESM.docx]

**Does long-term care insurance reduce the disability of middle-aged and older adults?** **Evidence from China**

Suppmentary appendix

| Table 1a List of the first pilot cities of the LTCI | | |
| --- | --- | --- |
| **Name of city** | **Province** | **Insured personnel** |
| Chende | Hebei | participants of The Urban Employee Basic Medical Insurance (UEBMI) |
| Changchun | Shenyang | participants of UEBMI;  participants Urban Resident Basic Medical Insurance (URBMI) |
| Qiqihaer | Heilongjiang | participants of UEBMI |
| Shanghai | Shanghai | participants of UEBMI; participants of urban and rural residents Basic Medical Insurance (URRMI) who above 60 years old |
| Nantong | Jiangsu | participants of UEBMI; participants of URRMI |
| Suzhou | Jiangsu | participants of UEBMI; participants of URRMI |
| Ningbo | Zhejiang | participants of UEBMI |
| Anqing | Anhui | participants of UEBMI |
| Shangrao | Jiangxi | participants of UEBMI |
| Qingdao | Shandong | participants of UEBMI; participants of URRMI |
| Jingmen | Hubei | participants of UEBMI; participants of URRMI |
| Guangzhou | Guangdong | participants of UEBMI |
| Chongqing | Chongqing | participants of UEBMI |
| Chengdu | Sichuan | participants of UEBMI |
| Shihezi | Guangdong | participants of UEBMI; participants of URRMI |

*Data source：According to the material of pilot cities*

Table 1b List of the second pilot cities of the LTCI

| **Name of city** | **Province** | **Insured personnel** |
| --- | --- | --- |
| Shijingshan District | Beijing | participants of UEBMI; participants of URRMI |
| Tianjin | Tianjin | participants of UEBMI |
| Jinchen | Shanxi | participants of UEBMI |
| Hohhot | Inner Mongolia | participants of UEBMI; participants of URRMI |
| Panjin | Liaoning | participants of UEBMI |
| Fuzhou | Fujian | participants of UEBMI |
| Kaifang | Henan | participants of UEBMI |
| Xiangtan | Hunan | participants of UEBMI |
| Nanning | Guangxi | participants of UEBMI |
| Qianxi'Nan Buyei and Miao Autonomous Prefecture | Guizhou | participants of UEBMI |
| Kuiming | Yunnan | participants of UEBMI |
| Hanzhong | Shannxi | participants of UEBMI |
| Gannan Tibetan Autonomous Prefecture | Gansu | participants of UEBMI |
| Urumqi | Xinjiang | participants of UEBMI |

*Data source：According to the material of pilot cities*

Table 2a Descriptive statistics of variables in treated group

| Variables | 2011 | | |  | 2013 | | |  | 2015 | | |  | 2018 | | |
| --- | --- | --- | --- | --- | --- | --- | --- | --- | --- | --- | --- | --- | --- | --- | --- |
|  | mean | SD | N |  | mean | SD | N |  | mean | SD | N |  | mean | SD | N |
| IADL | 0.180 | 0.614 | 194 |  | 0.223 | 0.752 | 175 |  | 0.268 | 0.777 | 183 |  | 0.268 | 0.777 | 183 |
| ADL | 0.180 | 0.635 | 189 |  | 0.152 | 0.606 | 178 |  | 0.186 | 0.543 | 183 |  | 0.368 | 1.092 | 193 |
| FL | 1.010 | 1.288 | 194 |  | 0.854 | 1.222 | 178 |  | 0.820 | 1.312 | 183 |  | 1.425 | 1.707 | 193 |
| Residence | 0.268 | 0.444 | 194 |  | 0.265 | 0.443 | 178 |  | 0.273 | 0.447 | 183 |  | 0.268 | 0.444 | 193 |
| Living status | 0.907 | 0.291 | 194 |  | 0.830 | 0.377 | 178 |  | 0.835 | 0.372 | 183 |  | 0.804 | 0.398 | 193 |
| Basic medical insurance | 0.911 | 0.285 | 194 |  | 0.948 | 0.222 | 178 |  | 0.923 | 0.267 | 183 |  | 1.000 | 0.000 | 193 |
| Smoking | 4.481 | 9.501 | 194 |  | 0.898 | 3.794 | 178 |  | 4.626 | 9.921 | 183 |  | 6.350 | 10.437 | 193 |
| Drinking | 1.542 | 2.673 | 194 |  | 1.775 | 2.690 | 178 |  | 1.577 | 2.639 | 183 |  | 1.360 | 2.461 | 193 |
| Chronic disease | 1.167 | 1.243 | 194 |  | 1.337 | 1.336 | 178 |  | 1.828 | 1.596 | 183 |  | 2.148 | 1.666 | 193 |
| Depression | 6.432 | 5.744 | 194 |  | 6.630 | 5.04 | 178 |  | 5.616 | 5.514 | 183 |  | 9.090 | 4.552 | 193 |

| Table 2b Descriptive statistics of variables in control group | | | | | | | | | | | | | | | |
| --- | --- | --- | --- | --- | --- | --- | --- | --- | --- | --- | --- | --- | --- | --- | --- |
| Variables | 2011 | | |  | 2013 | | |  | 2015 | | |  | 2018 | | |
|  | mean | SD | N |  | mean | SD | N |  | mean | SD | N |  | mean | SD | N |
| IADL | 0.378 | 0.942 | 10186 |  | 0.355 | 0.866 | 7735 |  | 0.412 | 0.963 | 9747 |  | 0.374 | 0.902 | 7134 |
| ADL | 0.297 | 0.885 | 10128 |  | 0.311 | 0.846 | 7799 |  | 0.387 | 0.968 | 9841 |  | 0.370 | 0.954 | 7529 |
| FL | 1.162 | 1.453 | 10224 |  | 1.402 | 1.583 | 7799 |  | 1.393 | 1.632 | 9841 |  | 1.591 | 1.720 | 7529 |
| Residence | 0.641 | 0.480 | 10224 |  | 0.649 | 0.477 | 7833 |  | 0.653 | 0.476 | 9884 |  | 0.641 | 0.480 | 7529 |
| Living status | 0.893 | 0.309 | 10224 |  | 0.880 | 0.325 | 7833 |  | 0.871 | 0.336 | 9884 |  | 0.795 | 0.404 | 7529 |
| Basic medical insurance | 0.934 | 0.248 | 10224 |  | 0.966 | 0.181 | 7833 |  | 0.914 | 0.280 | 9884 |  | 0.975 | 0.157 | 7529 |
| Smoking | 4.397 | 9.584 | 10224 |  | 2.601 | 7.471 | 7833 |  | 4.790 | 9.675 | 9884 |  | 7.194 | 18.400 | 7529 |
| Drinking | 0.952 | 2.126 | 10224 |  | 0.933 | 2.101 | 7833 |  | 1.269 | 2.375 | 9884 |  | 0.994 | 2.128 | 7529 |
| Chronic disease | 1.298 | 1.269 | 10224 |  | 1.492 | 1.350 | 7833 |  | 1.919 | 1.524 | 9884 |  | 2.300 | 1.690 | 7529 |
| Depression | 8.330 | 6.290 | 10224 |  | 8.064 | 5.818 | 7833 |  | 7.997 | 6.349 | 9884 |  | 9.962 | 5.559 | 7529 |

Table 2c The number of older adults who had ADL, IADL and FL scores of more than 1 in treated group

| Variables | 2011 | 2013 | 2015 | 2018 |
| --- | --- | --- | --- | --- |
| IADL | 19 | 19 | 27 | 27 |
| ADL | 19 | 18 | 23 | 27 |
| FL | 100 | 82 | 73 | 110 |

Table 3a PSM balance test (IADL)

| Variables | Unmatched/matched | Mean | | Bias% | t test | |
| --- | --- | --- | --- | --- | --- | --- |
|  |  | Treated | Control |  | t | p> \| t \| |
| Living status | Unmatched | 0.780 | 0.838 | -14.8 | -1.83 | 0.067 |
|  | Matched | 0.779 | 0.762 | 4.4 | 0.34 | 0.738 |
| Residence | Unmatched | 0.345 | 0.205 | 31.7 | 4.02 | 0.000 |
|  | Matched | 0.345 | 0.333 | 2.8 | 0.21 | 0.832 |
| Basic medical insurance | Unmatched | 0.992 | 0.968 | 18.0 | 1.65 | 0.099 |
|  | Matched | 0.992 | 0.992 | 0.0 | 0.00 | 1.000 |
| Smoke | Unmatched | 5.404 | 4.622 | 6.5 | 0.66 | 0.508 |
|  | Matched | 5.404 | 5.616 | -1.8 | -0.16 | 0.876 |
| Drink | Unmatched | 1.074 | 0.967 | 4.9 | 0.58 | 0.559 |
|  | Matched | 1.074 | 0.951 | 5.7 | 0.46 | 0.642 |
| Chronic disease | Unmatched | 2.022 | 1.873 | 9.7 | 1.10 | 0.270 |
|  | Matched | 2.022 | 2.260 | -15.5 | -1.22 | 0.223 |
| Depression | Unmatched | 8.802 | 8.893 | -1.7 | -0.18 | 0.855 |
|  | Matched | 8.802 | 9.074 | -5.2 | -0.44 | 0.664 |
| Age | Unmatched | 61.838 | 60.619 | 14.9 | 1.63 | 0.104 |
|  | Matched | 61.838 | 62.201 | -4.4 | -0.37 | 0.712 |
| Gender | Unmatched | 0.552 | 0.623 | -14.5 | -1.70 | 0.089 |
|  | Matched | 0.552 | 1.583 | -6.5 | -0.53 | 0.597 |
| Education | Unmatched | 1.221 | 1.133 | 20.6 | 2.66 | 0.008 |
|  | Matched | 1.221 | 1.208 | 2.9 | 0.21 | 0.833 |

Table 3b PSM balance test (ADL)

| Variables | Unmatched/matched | Mean | | Bias% | t test | |
| --- | --- | --- | --- | --- | --- | --- |
|  |  | Treated | Control |  | t | p> \|t\| |
| Living status | Unmatched | 0.771 | 0.835 | -16.0 | -2.02 | 0.043 |
|  | Matched | 0.771 | 0.785 | -3.6 | -0.29 | 0.774 |
| Residence | Unmatched | 0.364 | 0.207 | 35.4 | 4.58 | 0.000 |
|  | Matched | 0.364 | 0.383 | -4.3 | -0.33 | 0.743 |
| Basic medical insurance | Unmatched | 0.993 | 0.967 | 18.3 | 1.69 | 0.091 |
|  | Matched | 0.993 | 0.993 | 0.0 | 0.00 | 1.000 |
| Smoke | Unmatched | 5.250 | 4.654 | 5.0 | 0.51 | 0.607 |
|  | Matched | 5.250 | 7.989 | -22.9 | -0.54 | 0.591 |
| Drink | Unmatched | 1.057 | 0.966 | 4.2 | 0.51 | 0.613 |
|  | Matched | 1.057 | 1.057 | 0.0 | 0.00 | 1.000 |
| Chronic disease | Unmatched | 1.993 | 1.870 | 8.0 | 0.92 | 0.356 |
|  | Matched | 1.993 | 2.095 | -6.7 | -0.53 | 0.595 |
| Depression | Unmatched | 8.757 | 8.922 | -3.2 | -0.34 | 0.737 |
|  | Matched | 8.757 | 8.348 | 7.8 | 0.69 | 0.491 |
| Age | Unmatched | 61.950 | 60.677 | 15.6 | 1.72 | 0.085 |
|  | Matched | 61.950 | 61.964 | -0.2 | -0.02 | 0.988 |
| Gender | Unmatched | 0.564 | 0.622 | -11.8 | -1.40 | 0.160 |
|  | Matched | 0.564 | 0.588 | -4.8 | -0.40 | 0.688 |
| Education | Unmatched | 1.236 | 1.134 | 23.6 | 3.11 | 0.002 |
|  | Matched | 1.236 | 1.224 | 2.8 | 0.20 | 0.840 |

Table 3c PSM balance test (FL)

| Variables | Unmatched/matched | Mean | | Bias% | t test | |
| --- | --- | --- | --- | --- | --- | --- |
|  |  | Treated | Control |  | t | p> \| t \| |
| Living status | Unmatched | 0.771 | 0.835 | -16.0 | -2.02 | 0.043 |
|  | Matched | 0.771 | 0.786 | -3.6 | -0.29 | 0.774 |
| Residence | Unmatched | 0.364 | 0.207 | 35.4 | 4.58 | 0.000 |
|  | Matched | 0.364 | 0.383 | -4.3 | -0.33 | 0.743 |
| Basic medical insurance | Unmatched | 0.993 | 0.967 | 18.3 | 1.69 | 0.091 |
|  | Matched | 0.993 | 0.993 | 0.0 | 0.00 | 1.000 |
| Smoke | Unmatched | 5.250 | 4.654 | 5.0 | 0.51 | 0.607 |
|  | Matched | 5.250 | 7.989 | -22.9 | -0.54 | 0.591 |
| Drink | Unmatched | 1.057 | 0.966 | 4.2 | 0.51 | 0.613 |
|  | Matched | 1.057 | 1.057 | 0.0 | 0.00 | 1.000 |
| Chronic disease | Unmatched | 1.993 | 1.870 | 8.0 | 0.92 | 0.356 |
|  | Matched | 1.993 | 2.095 | -6.7 | -0.53 | 0.595 |
| Depression | Unmatched | 8.757 | 8.922 | -3.2 | -0.34 | 0.737 |
|  | Matched | 8.757 | 8.348 | 7.8 | 0.69 | 0.491 |
| Age | Unmatched | 61.950 | 60.677 | 15.6 | 1.72 | 0.085 |
|  | Matched | 61.950 | 61.964 | -0.2 | -0.02 | 0.988 |
| Gender | Unmatched | 0.564 | 0.622 | -11.8 | -1.40 | 0.160 |
|  | Matched | 0.564 | 0.588 | -4.8 | -0.40 | 0.688 |
| Education | Unmatched | 1.236 | 1.134 | 23.6 | 3.11 | 0.002 |
|  | Matched | 1.236 | 1.224 | 2.8 | 0.20 | 0.840 |
